# Sulfate, nitrate and blood pressure – An EPIC interaction between sulfur and nitrogen

**DOI:** 10.1016/j.phrs.2017.06.006

**Published:** 2017-08

**Authors:** Gunter G. Kuhnle, Robert Luben, Kay-Tee Khaw, Martin Feelisch

**Affiliations:** a Department of Food & Nutritional Sciences, University of Reading, Reading, RG6 6UA, UK; b Department of Public Health and Primary Care, University of Cambridge, UK; c Clinical & Experimental Sciences, Medicine and NIHR Southampton Biomedical Research Centre, University of Southampton and University Hospital Southampton NHS Foundation Trust, Southampton, SO16 6YD, UK

**Keywords:** Bioactivation, Microbial flora, Nitric oxide, Nitrite, Nitrate, Hydrogen sulfide

## Abstract

Nitrate (NO_3_^−^)-rich foods such as green leafy vegetables are not only part of a healthy diet, but increasingly marketed for primary prevention of cardiovascular disease (CVD) and used as ergogenic aids by competitive athletes. While there is abundant evidence for mild hypotensive effects of nitrate on acute application there is limited data on chronic intake in humans, and results from animal studies suggest no long-term benefit. This is important as nitrate can also promote the formation of nitrosamines. It is therefore classified as ‘*probably carcinogenic to humans'*, although a beneficial effect on CVD risk might compensate for an increased cancer risk.

Dietary nitrate requires reduction to nitrite (NO_2_^−^) by oral commensal bacteria to contribute to the formation of nitric oxide (NO). The extensive crosstalk between NO and hydrogen sulfide (H_2_S) related metabolites may further affect nitrate’s bioactivity. Using nitrate and nitrite concentrations of drinking water − the only dietary source continuously monitored for which detailed data exist − in conjunction with data of >14,000 participants of the EPIC-Norfolk study, we found no inverse associations with blood pressure or CVD risk. Instead, we found a strong interaction with sulfate (SO_4_^2−^). At low sulfate concentrations, nitrate was inversely associated with BP (−4 mmHg in top quintile) whereas this was reversed at higher concentrations (+3 mmHg in top quintile).

Our findings have a potentially significant impact for pharmacology, physiology and public health, redirecting our attention from the oral microbiome and mouthwash use to interaction with sulfur-containing dietary constituents. These results also indicate that nitrate bioactivation is more complex than hitherto assumed. The modulation of nitrate bioactivity by sulfate may render dietary lifestyle interventions aimed at increasing nitrate intake ineffective and even reverse potential antihypertensive effects, warranting further investigation.

## Graphical abstract

**Figure:**
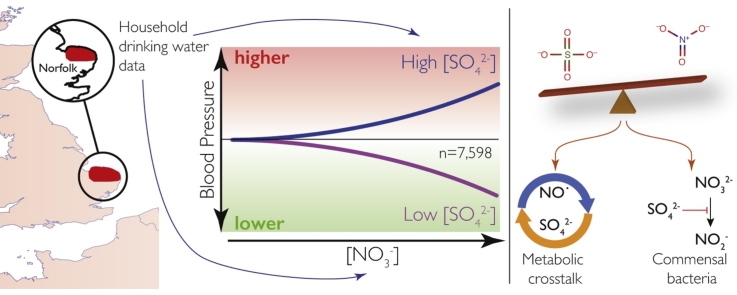

## 1 Introduction

A decade ago inorganic nitrate (NO_3_^−^) was reported to lower blood pressure (BP) in healthy volunteers [1]. Together with its capacity to reduce the oxygen cost of exercise reported earlier [2] these observations triggered a change in our perception of the risk/benefit ratio of this simple anion. Despite unanimous confirmation of these beneficial effects on acute and sub-acute application by many different groups since, no data exist for long-term intake of nitrate in humans. Recent animal studies did not show persistent anti-hypertensive effects following chronic administration of higher nitrate doses, possibly because of either compensatory down-regulation of eNOS or enhanced renal excretion [3], [4]. Moreover, considerable controversy remains regarding the long-term health effects of dietary nitrate and nitrite (NO_2_^−^) [5], which are classified by IARC as *probably carcinogenic to humans*, due to their potential to form *N*-nitrosamines [6]. This classification is in stark contrast to the use of rather astonishing doses of nitrate in medicine of the past [7] and the current development of nitrite (intravenously and in nebulised form for inhalation) to treat pulmonary arterial hypertension, heart failure and other ailments.

The main dietary sources of nitrate (and nitrite) are vegetables, processed meat and drinking water. Nitrate is also formed by reaction of endogenously produced nitric oxide (NO) with oxygenated hemoglobin in red blood cells; it enjoys a relatively long half-life in blood (5–7 h in humans) due to renal reabsorption. About a fifth to a quarter of the circulating amounts of nitrate are actively secreted into saliva; here, dietary and endogenously produced nitrate is reduced to nitrite by facultative anaerobic bacteria that are part of our commensal oral microflora. Upon swallowing, nitrite ends up in the stomach where the protonated form (nitrous acid, HNO_2_) regenerates NO, contributing to the BP-lowering effects of acutely administered nitrate [8].

Nitrate-rich foods including green leafy vegetables are considered to be part of a healthy diet. In the form of beetroot juice nitrate is increasingly promoted as ergogenic aid and used by competitive athletes and sports enthusiasts. Nitrate is also actively marketed for the primary prevention of cardiovascular diseases, despite utter lack of evidence and data on chronic intake. This development would seem to call for a careful reassessment of nitrate’s risk/benefit profile: a small but significant reduction of BP at population level may well outweigh its purported risks, but the absence of such an effect − or even an increased BP − could have severe consequences by increasing e.g. the risk of stroke or cardiovascular mortality.

## 2 How to tackle a decade-long controversy?

Investigating long-term associations with BP in observational studies is difficult because of the challenges associated with accurately assessing dietary exposure, due in part to marked geographical and seasonal variation in foods and further complicated by extensive microbial metabolism. The only source of intake regularly consumed for which reliable longitudinal data exists is drinking water (used for the preparation of tea/coffee and cooking, contributing to 30–50% of overall intake [9]) as its quality is closely monitored. The day-to-day variability of drinking water nitrate and nitrite is small and therefore provides a constant background onto which intake from other sources is added. Given the high variability of all other sources, it is therefore a suitable surrogate marker for long-term exposure.

We therefore used household-level municipal water records to investigate potential associations between drinking water nitrite/nitrate concentrations and BP in 15,549 participants of EPIC-Norfolk [10]. Because of emerging evidence for ample crosstalk between NO and sulfur metabolites [11] we also investigated whether there was an interaction with sulfate (SO_4_^2−^).

## 3 Combining EPIC—Norfolk cohort data with routine drinking water analyses − main findings

**Fig. 1:**
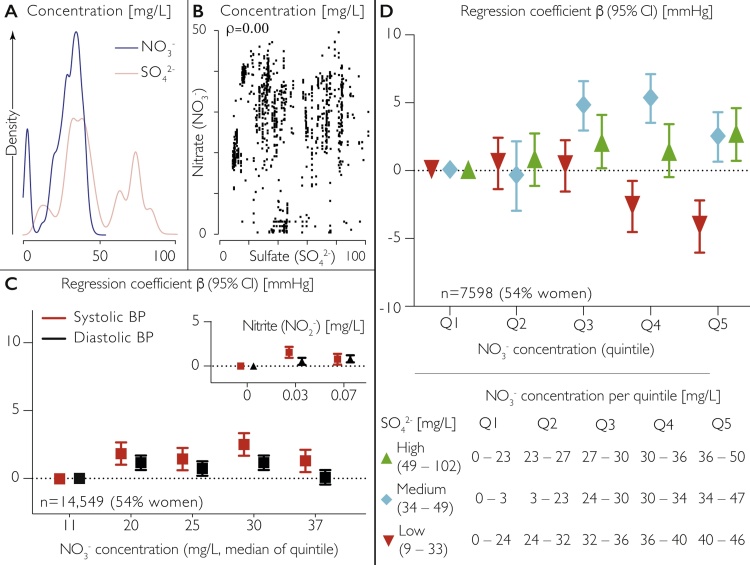
Interaction between drinking water nitrate and sulfate concentration and blood pressure (BP). (**A**) Relative distribution of and (**B**) correlation between drinking water nitrate and sulfate concentrations in 4112 women and 3486 men of EPIC Norfolk for whom all concentration data were available. **C**: Association between drinking water nitrate and nitrite concentrations and blood pressure in 7903 women and 6646 men of EPIC Norfolk for whom nitrate and nitrite concentration data were available. Regression coefficients β (95% CI) by quintile/tertile of concentration, adjusted by age, sex, menopausal status, plasma vitamin C, smoking status, physical activity and social class. **D** Interaction between drinking water nitrate and sulfate concentrations and systolic blood pressure in 4112 women and 3486 men of EPIC Norfolk (adjusted by age, sex, menopausal status, plasma vitamin C, smoking status, physical activity and social class). Regression coefficient β (95% CI) by quintile of drinking water nitrate concentration ([NO_3_^−^]), stratified by tertile of sulfate concentration ([SO_4_^2−^]). Data for participants’ water supply at time of health check obtained from Anglian Water Services, Huntington, UK.

Drinking water nitrite concentrations were below 0.5 mg/L for all participants. The ranges of nitrate and sulfate concentrations were considerably larger (Fig. 1A), but there was no correlation between the concentrations of these anions (Fig. 1B). Unexpectedly, we found no inverse association between nitrate or nitrite concentrations and BP at baseline; if anything, the associations were mildly positive (Fig. 1C). Likewise, after an average of 15 years of follow-up, we did not find inverse associations with CVD risk (HR 1.02; 95% CI 0.99; 1.04 per 10 mg/L nitrate concentration increase, adjusted for sex, BMI, physical activity, smoking status, plasma vitamin C and social class). There was also no direct association between drinking water sulfate and BP in 7598 participants for whom this data was available (not shown). However, we found a statistically significant interaction (p < 0.01) between nitrate and sulfate concentrations: at low sulfate concentrations, nitrate was inversely associated with BP (−4 mmHg in top quintile) whereas this association was reversed at higher concentrations (+3 mmHg in top quintile) (Fig. 1D). These findings suggest that the long-term effects of nitrate on BP are influenced by concomitant sulfate intake and that nitrate bioactivation is more complex than hitherto envisaged.

## 4 Possible mechanisms contributing to this interaction

The mechanism of this interaction is currently unknown and requires further study. One possible explanation may be related to the crosstalk between sulfur and nitrogen metabolic pathways. Higher dietary sulfate intake may shift the balance between transsulfuration and methionine recycling towards the remethylation of homocysteine, thereby leading to an alteration of global redox status while attenuating endogenous NO formation following inhibition of endothelial nitric oxide synthase (eNOS) via S-adenosylmethionine-mediated asymmetric dimethylarginine formation [12]. An alternative explanation involves competition at the level of host/microbial co-metabolism. This might conceivably include reduction of sulfate to sulfite (SO_3_^2−^) or hydrogen sulfide (H_2_S/HS^−^) by sulfate-reducing gut bacteria, followed by either scavenging of NO and/or interference with NO_2_^−^ metabolism in the host tissues. Alternatively, sulfate may compete with bacterial nitrate reduction to nitrite in the oral cavity, as described for the microbial flora in the large intestine. [13] Yet another possibility is that the chronic intake of different levels of nitrate and sulfate modulates the composition of the gut microbial flora such that it alters the profile of fermentation products formed in the gut, including those released into the systemic circulation and capable of modulating vascular tone. Whatever the mechanism, we conclude that variations in dietary sulfate intake have the potential to affect BP by modulating the effects of nitrate on the cardiovascular system.

## 5 Potential implications for pharmacology, physiology and public health

These findings have a potentially significant impact on public health and may also explain some of the variability observed in hemodynamic effects with (nitrite and) nitrate in pharmacological studies. Nitrate-rich foods, often exceeding the *acceptable daily intake* (3.7 mg/kg body weight) for nitrate, are increasingly marketed for the primary prevention of hypertension and cardiovascular diseases, despite the lack of robust long-term data and animal experimental results suggesting no long-term beneficial effects. The significance of the oral microbiome for the efficacy of nitrate is well-known, in particular the potential of antibacterial mouthwashes to abolish its BP-lowering effects [14]. Yet in contrast to mouthwash use, sulfate intake is more difficult to control for consumers; little is known about the sulfate content of food, and drinking water sulfate is not as tightly controlled as that of nitrate and nitrite. An interaction between sulfate and nitrate, as shown here, may render dietary lifestyle changes or interventions aimed at increasing nitrate intake ineffective and possibly even reverse potential antihypertensive effects of nitrate.

## Conflicts of interest

The authors declare no conflict of interest.
